# Enhanced Healing of Rat Calvarial Defects with MSCs Loaded on BMP-2 Releasing Chitosan/Alginate/Hydroxyapatite Scaffolds

**DOI:** 10.1371/journal.pone.0104061

**Published:** 2014-08-01

**Authors:** Xiaoning He, Yang Liu, Xue Yuan, Li Lu

**Affiliations:** 1 Department of Stomatology, the 4^th^ Affiliated Hospital of China Medical University, Shenyang, Liaoning, China; 2 Department of Oral and Maxillofacial Surgery, School of Stomatology, China Medical University, Shenyang, Liaoning, China; 3 Department of Oral Biology, The State University of New York at Buffalo, Buffalo, New York, United States of America; Ohio State University, United States of America

## Abstract

In this study, we designed a chitosan/alginate/hydroxyapatite scaffold as a carrier for recombinant BMP-2 (CAH/B2), and evaluated the release kinetics of BMP-2. We evaluated the effect of the CAH/B2 scaffold on the viability and differentiation of bone marrow mesenchymal stem cells (MSCs) by scanning electron microscopy, MTS, ALP assay, alizarin-red staining and qRT-PCR. Moreover, MSCs were seeded on scaffolds and used in a 8 mm rat calvarial defect model. New bone formation was assessed by radiology, hematoxylin and eosin staining 12 weeks postoperatively. We found the release kinetics of BMP-2 from the CAH/B2 scaffold were delayed compared with those from collagen gel, which is widely used for BMP-2 delivery. The BMP-2 released from the scaffold increased MSC differentiation and did not show any cytotoxicity. MSCs exhibited greater ALP activity as well as stronger calcium mineral deposition, and the bone-related markers Col1α, osteopontin, and osteocalcin were upregulated. Analysis of in vivo bone formation showed that the CAH/B2 scaffold induced more bone formation than other groups. This study demonstrates that CAH/B2 scaffolds might be useful for delivering osteogenic BMP-2 protein and present a promising bone regeneration strategy.

## Introduction

Craniofacial bone defects associated with trauma, pathology and fracture nonunion represent a significant clinical problem [1], [2]. Autograft is the current gold standard treatment for bone grafting; however, it is limited by available volume of graft material, donor site morbidity and unpredictable bone resorption [3], [4]. Allografts are good alternatives to bridge defects, but risk of disease transmission and adverse host immune reactions limit the use of allograft. Hence, improved strategies are urgently needed to better treat craniofacial bone defects [5], [6].

Tissue engineering is a relatively new method to repair damaged bone. In bone tissue engineering, porous scaffolds serve as vehicles to deliver and retain cells at a specific site, guide new bone formation into desired shapes, maintain space and prevent soft tissue prolapse in the bony lesion. Therefore, the scaffold materials must be biocompatible, osteoconductive, and have enough mechanical strength to provide structural support [7], [8].

Recently, chitosan has garnered substantial interest in bone tissue engineering [9]. Chitosan is a natural cationic polymer that is biodegradable, biocompatible, non-antigenic and biofunctional. It has been studied as a useful biomaterial in tissue engineering because its hydrophilic surface promotes cell adhesion and proliferation [10], [11]. However, a pure chitosan scaffold is fragile and lacks the bioactivity to induce hard tissue formation, which limits its application in bone tissue engineering.

Hydroxyapatite (HA) has also attracted a great deal of attention recently [12], [13]. HA has been widely used in medicine because it is osteoconductive and has excellent biological affinity with bony tissue [14], possessing a similar chemical composition and structure as the mineral phase of bones. As a result, HA is accepted as a bioactive scaffold material for guided bone regeneration [15]. It has been reported that when HA is combined with chitosan for bone tissue engineering, it can increase the bioactivity and mechanical properties of the materials [16], [17].

Biological factors such as growth factors and cells are also typically required to effectively repair challenging bone defects. Bone morphogenetic protein-2 (BMP-2) has been shown to be a promising therapeutic agent promoting bone regeneration when delivered locally, but it has been demonstrated that adenovirus mediated BMP gene therapy can lead to harmful side effects such as tumorigenesis [18]. To provide specific and optimal biological activity, it is essential to design an appropriate carrier that retains BMP-2 and releases it slowly for bone formation. Several materials have already been evaluated as BMP carriers, such as collagen and other inorganic materials [19], [20], [21]. Although these materials can induce bone formation at orthotopic sites, they still have disadvantages such as the potential risk of immunogenicity, fragility, etc [22], [23]. Therefore, we have focused on alginate for BMP delivery. Alginate is a natural anionic polysaccharide that is already approved by the FDA for human use as a wound dressing [24], [25]. It has been widely used in cell culture and drug delivery, and its uses in long-term culture of osteocytes have been extensively documented [26], [27]. It has been reported that alginate and chitosan molecules form a polyelectrolyte complex through ionic interactions, and Ca+ ^2^ crosslinking reactions were also found in alginate/HA scaffolds as a result of divalent cations [11], [28].

Mesenchymal stem cells (MSCs) are a population of multipotent marrow-derived cells that are easily expanded in culture and differentiate into cells with an osteogenic phenotype. Implantation of MSCs has the potential to enhance healing of damaged bone. Therefore, we prepared CAH/B2 scaffolds through in situ co-precipitation and freeze drying, and evaluated the efficacy of the porous scaffolds for critical sized calvarial defect repair in rats. Our hypothesis was that when combined with MSCs, the CAH/B2 scaffold would provide greater defect closure and bone deposition compared to other groups, and inclusion of BMP-2 treatments would further augment defect closure and bone deposition.

## Materials and Methods

### Scaffold fabrication

CAH complex scaffolds with a calculated mass ratio of chitosan/alginate/HA = 1.25∶1.25∶1 were prepared through in situ co-precipitation [17], [29]. Briefly, 1.0 g of chitosan was dissolved in 2 wt% acetic acid solution under agitation, and 1 g of alginate powder was dissolved and thoroughly mixed in 25 mL of double distilled water. BMP-2 was loaded into the alginate solutions and agitated at room temperature; the final concentration of BMP-2 in this system was 200 ng/mL. Then 80 mL of 0.1 mol/L CaCl_2_ solution and 48 mL of 0.1 mol/L KH_2_PO_4_ solution were added to the aqueous chitosan and alginate solutions, respectively. The alginate solution was mixed with the chitosan solution under constant stirring in a blender for 1 h at 4°C. The solution was adjusted to pH 7.4 by adding acetic acid drop-wise, then placed into molds and frozen at −20°C. Then the samples were lyophilized in a freeze dryer at −80°C for 24 h. The dried samples were cross-linked with a 1% CaCl_2_ solution for 15 min and then immersed in distilled water to remove unbound CaCl_2_. After immersion, the samples were washed three times and then freeze-dried at −80°C for 24 h to obtain the BMP-2-loaded CAH composite scaffolds (CAH/B2).

### Scaffold characterization

The morphologies, pore configuration, and pore size of the CAH composite scaffolds were investigated using scanning electron microscopy as described [30]. The porosity and density were measured by the liquid displacement method [31]. The crystal structures were determined by x-ray diffraction (XRD) using anX-ray diffractometer with CuKα radiation(λ = 1.5418 Å) at room temperature, and the Jade 5 program was utilized for this analysis.

### In vitro BMP-2 release study from CAH scaffolds

CAH/B2 scaffolds were prepared as previously described. Type I collagen gel (Col-gel), which is widely used as a drug delivery system, was employed as a control for BMP-2 delivery. Col-gel was prepared from an acid solubilized type I collagen stock solution (BD Biosciences). According to the recommendation of the manufacturer, the stock solution was adjusted to a final concentration of 3 mg/ml of collagen solution containing 0.2 mg/mL BMP-2 (Col/B2). The CAH/B2 scaffolds were placed in containers with 1 mL of PBS (pH 7.4) at 37°C. At the designated time points, all of the supernatant was removed for sampling and replaced with fresh PBS [32]. The BMP-2 content of the supernatant was determined by the ELISA method. The optical density of each well was determined using a microplate reader at an optical density of 450 nm.

### Cell Preparation

Animal procedures were conducted in accordance with the protocol approved by China Medical University's Animal Care and Use Committee. Sprague-Dawley rats (6–8 weeks old) were euthanized by CO_2_. The rat femurs and tibias were dissected in a sterile hood. Metaphyses were resected from both ends and bone marrow was collected. The MSC were expanded according to procedures previously described [33]. The primary MSC were maintained in complete media containing α-MEM and 10% FBS. Cells were cultured in 100-mm culture dishes in a humidified, mixed environment at 37°C and 5% CO_2_.

### Cell proliferation and viability

MSCs were cultured in α-MEM with 10% FBS containing the BMP-2 released from the CAH/B2 scaffolds. When osteoblast differentiation was to be induced, 50 µg/mL of ascorbic acid and 5 mM β-glycerophosphate were added, and the culture media was changed every 3 days. To investigate the cytotoxicity of the CAH/B2 scaffolds to MSCs, the MTS assay was performed using the MTS cell viability kit (Promega) after 24, 48, 72 and 96 hr. Then the cells at different time points were processed in the MTS assay according to the user manual. OD values were measured at 490 nm using a microplate reader. CAH scaffold and plate group were set as controls.

### ALP activity assay and calcium mineral deposition

MSCs were cultured for 7, 14, and 21 days as described above. ALP activity was determined by using an ALP assay kit in accordance with the manufacturer's instructions (Sigma). ALP activity was normalized according to DNA content. DNA concentration was measured according to the method of Schneider [34]. To measure the level of calcium mineral deposition, alizarin-red staining (AR-S) was performed. After 3 weeks in culture, the cells were fixed with 70% ethanol, rinsed five times with deionized water, treated with 40 mM alizarin red solution for 10 min at pH 4.2, then washed for 15 min with PBS. Stained samples were treated with 10% cetylpyridinium chloride in 10 mM sodium phosphate for 15 min at room temperature. The AR-S concentration was determined by comparing it to an AR-S standard curve at an optical density of 540 nm.

### Quantitative reverse transcription-polymerase chain reaction (qRT-PCR)

Total RNA was isolated using Trizol reagent (Invitrogen). Reverse transcription of total RNA was carried out using the first strand synthesis system (Invitrogen). The synthesized cDNA was then used to perform real time-PCR [35]. PCR amplifications were performed using specific primers (Table 1) for analyzing the expression of osteocalcin (OCN), collagen type I, alpha 1 (Col1α1) and osteopontin (OPN). Real time PCR was performed on an ABI PRISM 7500 sequence detection system with SYBR GREEN PCR Master Mix. The PCR conditions were 94°C for 1 min followed by 95°C for 30 s and then 58°C for 40 s for a total of 35 cycles [30]. All of the reactions were run 3 times and were normalized to GAPDH. The relative differences in the PCR results were calculated by using the comparative Ct-method (2-ΔΔCT).

**Table 1:** Primer sequences of osteogenic markers.

	forward primer	reverse primer
OCN	5′- TCTTTCTCCTTTGCCTGGC -3′	5′- CACCGTCCTCAAATTCTCCC -3′
Col1a1	5′-GCAACAGTCGCTTCACCTACA -3′	5′-CAATGTCCAAGGGAGCCACAT-3′
OPN	5′-TATCCCGATGCCACAGATGA-3′	5′-TGAAACTCGTGGCTCTGATG-3′
GAPDH	5′- TGTGTCCGTCGTGGATCTGA -3′	5′-TTGCTGTTGAAGTCGCAGGAG -3′

### In vivo study

The in vivo experimental protocol was approved by China Medical University's Animal Care and Use Committee. Thirty male SD rats at 8 weeks of age were anesthetized with 5% isoflurane/O_2_ gas inspiration using a facial mask. Afterward the skin was prepped and sterilized with iodine and ethanol. An incision was made along the top of the skull, with full thickness retraction of the skin and periosteum, to expose the calvarium. Critical size defects (diameter = 8 mm) were created with a trephine bur under constant DPBS irrigation. Next, 1×10^6^ cells were seeded on scaffolds and implanted into the defect and then the soft tissue was sutured. After surgery, buprenornhine was used to minimize pain or discomfort and all rats were monitored for signs of infection. The animals were divided into five groups: group 1, blank control; group 2, CAH; group 3, CAH+MSCs (CAH+M); group 4, CAH/B2+MSCs (CAH/B2+M) and group 5, Col/B2+MSCs (Col/B2+M). At the end of the 12-week period, animals were euthanized using CO_2_ and the calvarial bones were harvested for further testing.

### Analysis of bone regeneration

Bone mineral density (BMD, g/cm^2^) was determined using a bone densitometer. The LUNAR PIXImus program was used to analyze the scanned data. A total of six samples per group were analyzed. On the computerized scan plots, five regions of interest (ROI) on each slide, from six slides per group, were selected to measure BMD in the defect area in each specimen; the average values were taken as the final result. For histological analysis, specimens were fixed, decalcified, embedded and sectioned into 5 µm thick sections. The sections were then stained with hematoxylin and eosin (H&E). Digital images of each slide were acquired using a digital camera mounted to a microscope. Newly formed bone areas in the total scaffold area were calculated manually using NIH ImageJ software.

### Statistical analyses

Statistical analysis was performed using the SPSS-17.0 program. Data were analyzed using one-way analysis of variance, and Tukey's HSD test was applied as a post hoc test if statistical significance was determined. Statistical significance for two groups was assessed using Student's t-test. The probability level at which differences were considered significant was P<0.05.

## Results

### Synthesis and Structure of scaffolds

**Figure 1:**
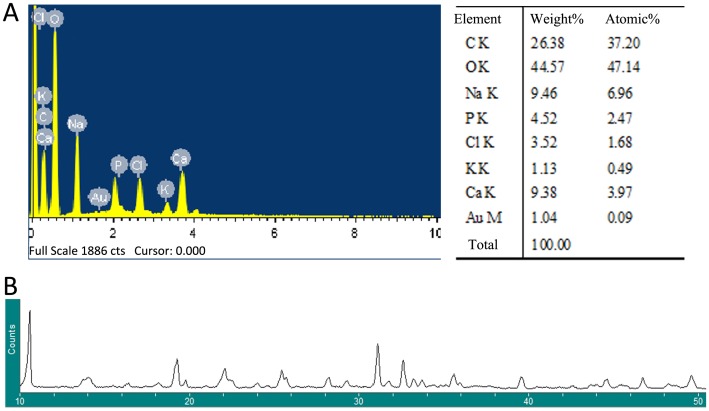
Characterization of CAH/B2 scaffolds. **A.** EDX analysis of CAH specimen. B. XRD pattern of CAH specimen.

**Figure 2:**
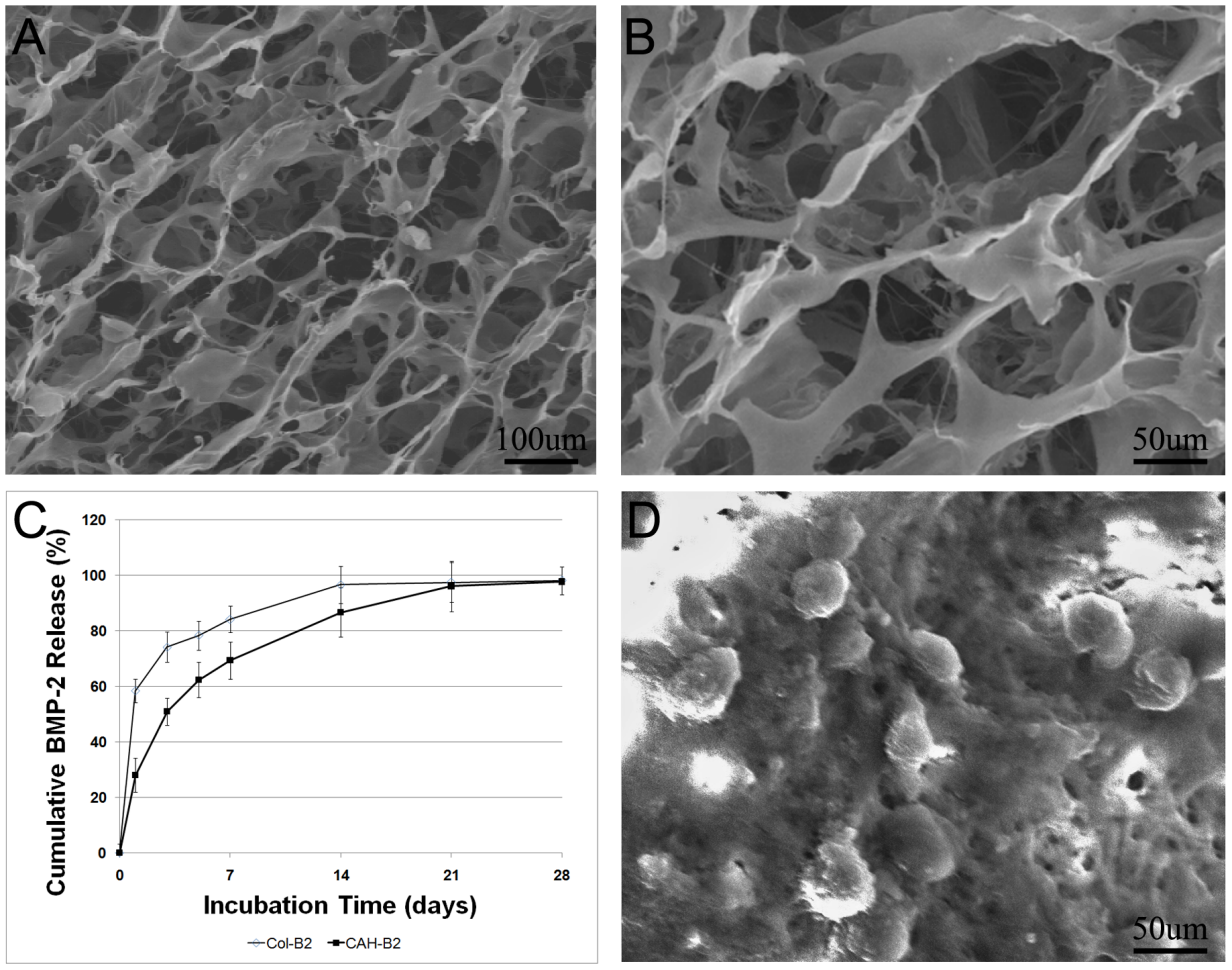
Physicochemical properties of CAH/B2 scaffolds. **A.** SEM view of CAH/B2 scaffold at a magnification of 100×, **B.** SEM view of CAH/B2 scaffold at a magnification of 200×. SEM analysis showed that micro-pores were generated in the CAH scaffold, the average pore diameter in the scaffold being 100–110 µm. **C.** In vitro release behavior of BMP-2 from the CAH scaffold. Col/B2 served as a control. The release velocity of BMP-2 was delayed in the CAH/B2 group compared with release from Col/B2. The cumulative BMP-2 released from the CAH/B2 and the Col/B2 groups almost reached a plateau at 21 and 14 days, respectively. **D.** SEM view of MSCs cultured on a CAH/B2 scaffold at a magnification of 100×.

The 3D porous structure of the CAH/B2 scaffold is produced by an situ co-precipitation method, followed by solvent sublimation through lyophilization. EDX analysis of the synthesized CAH specimens showed that the Ca/P ratio was 1.60, as illustrated in Figure 1A. Figure 1B shows the X-ray diffraction pattern of a CAH scaffold. After being incorporated into the CAH composite, the typical crystalline peaks of Chitosan (10.2°, 19.8°, 21.9°) alginate (13.4°, 21.4°) and HA(25.8°, 31.8°, 32.1°, 32.9°, 34°, 39.9°, 467.7°, 49.4°) still existed. But the peaks became broader and weaker as compared to the standard spectrum [36], [37], [38]. SEM analysis showed that micro-pores were generated in the CAH/B2 scaffold, the complex was highly porous and the average pore diameter was 100–110 µm (Figure 2A, B). Porosity is based on the presence of open pores and is related to properties such as the permeability and surface area of the porous structure. High porosity usually means a high surface area/volume ratio, thus favoring cell adhesion to the scaffold and promoting bone tissue regeneration [16], [39], [40], [41]. The porosity of the composite scaffolds was about 74.54%.

### In vitro BMP-2 release study

The in vitro release kinetics of BMP-2 from the scaffold were interpreted by the cumulative amount and percentage of the BMP-2 as a function of time. Figure 2C show the cumulative release of BMP-2 from the CAH/B2 scaffold. The release velocity of BMP-2 was delayed in the CAH/B2 group (64% within 7 days) compared with that in the Col/B2 group (84% within 7 days). The cumulative BMP-2 released from CAH/B2 and Col/B2 reached a near plateau at 21 and 14 days, respectively. These results suggest that the release velocity of the CAH/B2 scaffold exhibited a sustained release compared with that of the Col-gel over a 21 day period. ELISA results showed in the Col-B2 group, the concentration of BMP-2 was 12.65 ng/ml after 1 h, dropping to 7.9 ng/ml after 12 h, and 2.28 ng/ml after 7 days, while BMP-2 in the CAH group was 5.38 ng/ml after 1 h, was still around 4.59 ng/ml after 7 days, and was still maintained at 3.70 ng/ml at day 14.

### In vitro trials

**Figure 3:**
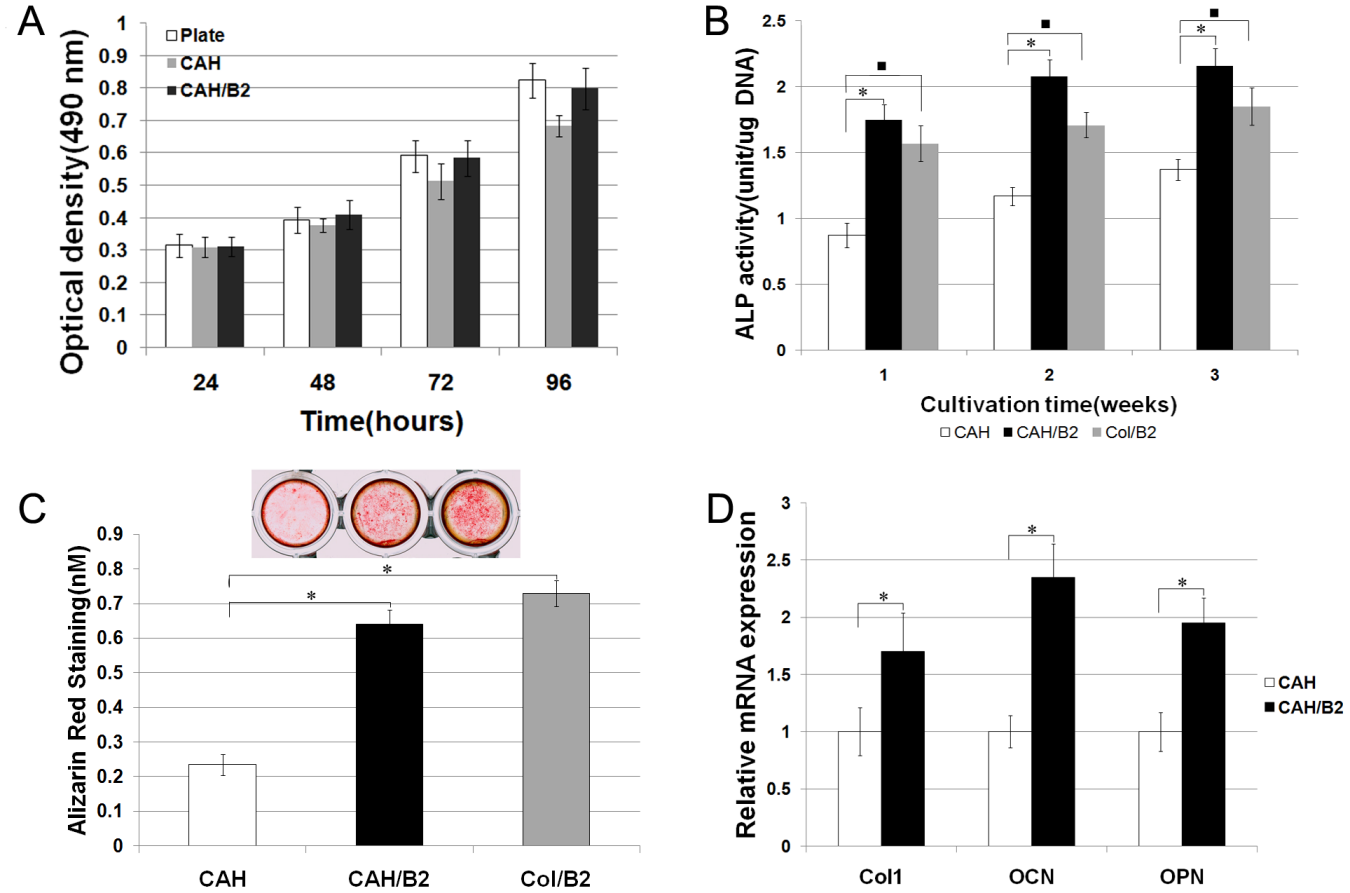
Biocompatibility and effects of the CAH/B2 scaffold on osteoblastic differentiation. **A.** MTS assay of MSCs cultured with CAH and CAH/B2 scaffolds. The cells were also cultured in plates as a control. Data represent the mean+SD of n = 5 samples. No statistically significant differences were seen between groups. **B.** Effects of CAH/B2 on in vitro ALP activity. ALP activity in the CAH/B2 group was higher than in the CAH group. One-way analyses of variance suggest that there are significant differences among three groups. The significant post hoc test results are identified by symbol *. N = 5. P<0.001: CAH vs. CAH/B2. **C.** The level of calcium deposition in 3-week cultures was evaluated by AR-S. The values indicated are means ± SD, (n = 5) p<0.005 as compared with that deposition in the CAH group. **D.** qRT-PCR analysis of osteoblast marker genes, showing that MSCs cultured on a CAH/B2 scaffold exhibited increased Col I, OCN and OPN gene expression. Data represent the mean + SD of n = 5 samples. P<0.05.

To determine whether the CAH/B2 scaffold affected MSCs' viability and proliferation, the MSCs were seeded on CAH/B2 scaffolds and cultured in vitro to verify that the scaffolds support MSCs in culture. SEM results indicated that the CAH/B2 scaffolds promoted a spherical morphology of MSC, which have been shown to maintain the multipotency and promote the differentiation efficiency of MSCs [42], [43] (Figure 2D). The biocompatibility of the CAH/B2 scaffolds was evaluated with the MTS assay. The cell population in the control increased continuously with time of cultivation, and the pattern of cell proliferation in the CAH/B2 group was similar to that in the control. These results show that the scaffolds exhibited no cytotoxic effects on the MSCs, and have good biocompatibility (Figure 3A).

### CAH scaffolds promote MSC osteoblast differentiation

The ALP activity and the level of calcium deposition are important considerations for evaluating osteoblast differentiation. ALP activity was measured after 1, 2, and 3 weeks in MSC cell cultures with the CAH/B2 scaffold. CAH and the Col/B2 were used as controls. As shown in Figure 3B, ALP activity was significantly increased in cells cultured with the CAH/B2 and Col/B2 compared with that of the CAH group. The level of calcium mineral deposition after 3 weeks in culture was investigated by AR-S. The results showed that calcium deposition in the CAH/B2 group was increased 2.7 fold compared with that of the CAH group (Figure 3C). Moreover, qRT-PCR showed that OCN, Col I, and OPN gene expression increased in the CAH/B2 group compared to the CAH group (Figure 3D).

### CAH scaffolds promote bone regeneration

**Figure 4:**
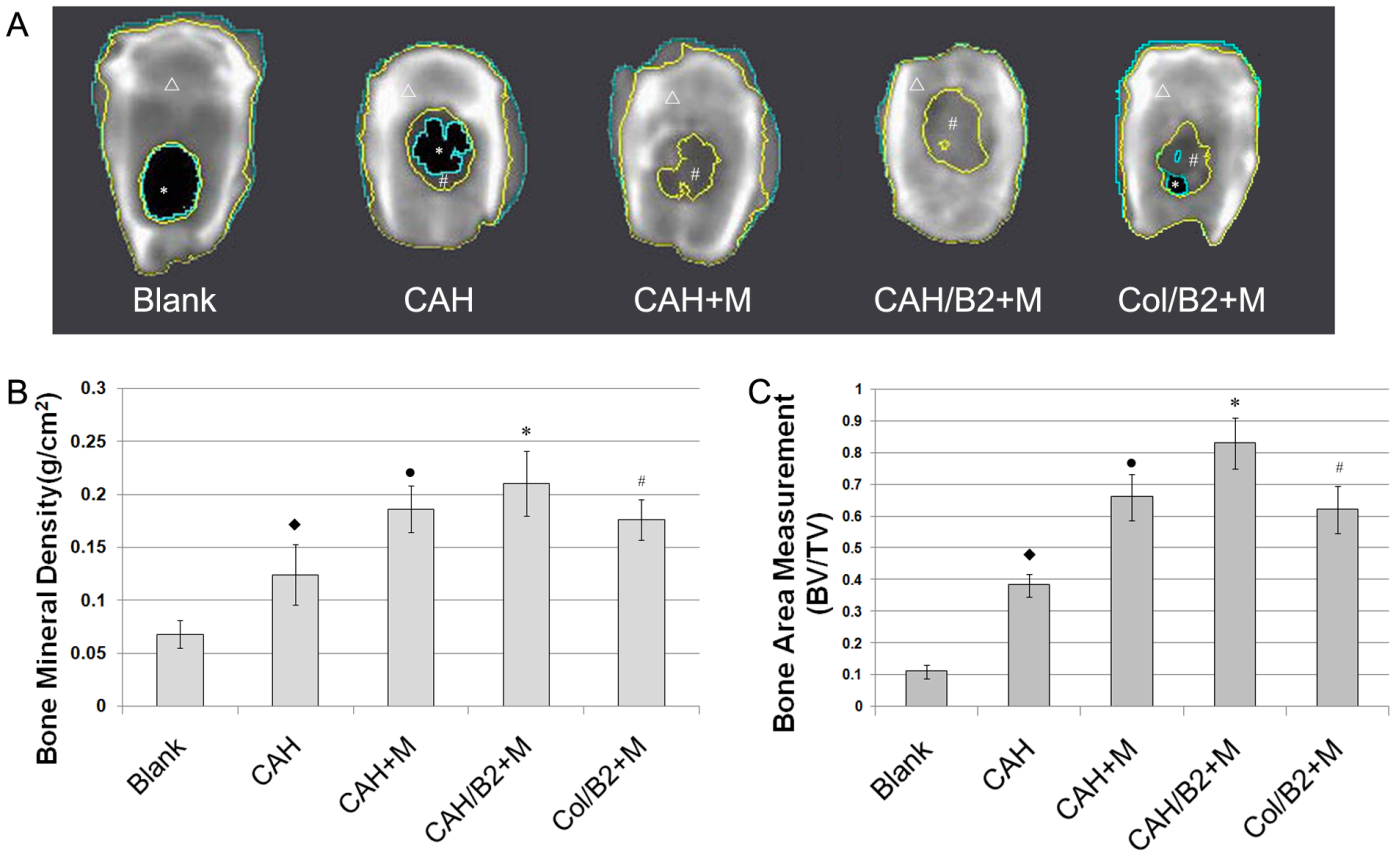
CAH/B2 scaffolds enhance bone regeneration in the rat critical-sized calvarial defect model. A. Image of calvarial bone from the LUNAR PIXImus system, 12 weeks after surgery. The areas of bone regeneration were labeled in different colors. A black area circled with a blue line (*) is a low density area. The dark gray area between inside blue line and inside yellow line (#) is an area of thin bone, the area between two yellow lines(▵) is considered an area of normal bone density, and the density in this area is close to that of normal bone tissue. B. Quantitative analysis of bone density. N =  6, *p<0.05:CAH/B2+M versus four other groups. • p<0.05: CAH+M versus CAH or blank. ♦p<0.05: CAH versus blank. C. Quantitative analysis of bone area in implanted region showed a significantly larger bone area within the CAH/B2+M group when compared with the other four groups. BV, bone area in the implant; TV, total implant area. N = 6, *p<0.05:CAH/B2+M versus four other groups. •p<0.05: CAH+M versus CAH or blank. ♦p<0.05: CAH versus blank.

**Figure 5:**
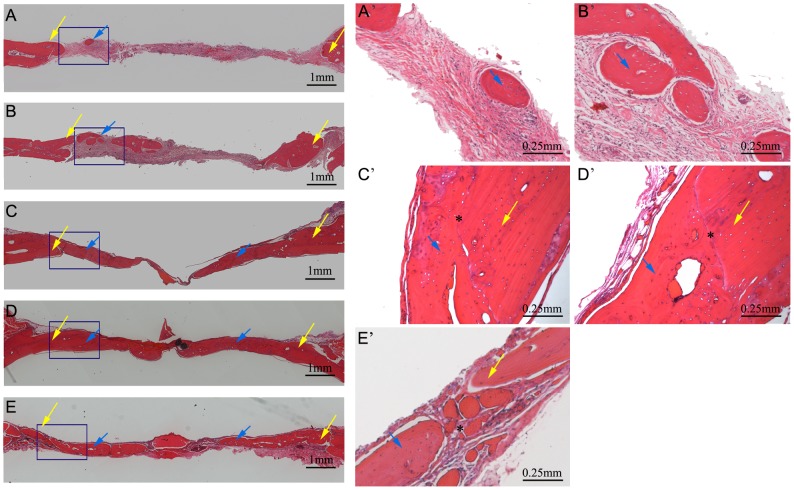
Histology analysis. Coronal sections through the midlines of defects are shown. (A–E): lower magnification, Bar = 1 mm, yellow arrow: new bone formed; (A′–E′): higher magnification, Bar = 0.25 mm. (A, A′): Blank; (B, B′): CAH; (C, C′): CAH+M; (D, D′): CAH/B2+M. (E, E′)Col/B2+M. Blue arrow: new bone, Yellow arrow: host bone, *:interface between new bone and host bone.

To evaluate the potential of the CAH scaffold for bone regeneration in vivo, an 8-mm defect was created in the calvarial bones of SD rats. Rats were divided into 5 groups: (1)empty defect (control), (2)CAH scaffold (CAH), (3)CAH scaffold+MSCs (CAH+M), (4)CAH/B2 scaffold+MSCs (CAH/B2+M) and (5)BMP-2 loaded Collagen type 1 gel+MSCs (Col/B2+M). The cranial bones were harvested 12 weeks after implantation and analyzed by radiology and histology. Quantitative analysis of BMD showed that the BMD in the CAH/B2+M group was significantly higher than in the other groups. The CAH/B2+M group exhibited robust osteogenic activity, with complete closure of bony defects. BMD in the CAH+M and Col/B2+M groups was also significantly higher than in the CAH and control groups; however, it was still much lower than in the CAH/B2+M group. Notably, the CAH group also showed higher BMD relative to the control group (Figure 4A,B). Furthermore, a histomorphometric analysis of histological slides also showed a significantly larger bone area within the CAH/B2+M group when compared with the other four groups (Figure 4C). Histological analysis confirmed these results, showing that, compared to the empty control (Figure 5A,A′), there were small regions of osteoid matrix within the interiors of the implants in the CAH group (Figure 5B, B′), indicating the CAH scaffold can stimulate new bone formation. Additionally, a larger amount of bone was formed in the CAH/B2+M group (Figure 5D,D′) compared to the CAH+M (Figure 5C,C′) and Col/B2+M groups (Figure 5E,E′), which induced partial bone defect healing. The CAH/B2+M group exhibited robust osteogenic activity, with complete coverage of defects with newly formed bone, and the interface between new bone and host bone showed a close union without any gaps.

## Discussion

To improve the healing of critical sized defects, to date, a major barrier has been the lack of sufficient integration of biomaterial design and engineered cells such as stem cells to promote bone regeneration [44], [45]. Although many studies use MSCs and scaffold minerals, this was the first that evaluated the combination of MSCs with a CAH/B2 scaffold in vivo. The results demonstrated the importance and efficiency of this system in bone regeneration, and highlighted the potential utility of this construct for bone repair and regeneration.

Scaffolds for bone tissue engineering must have a highly porous and interconnected pore structure. Greater porosity and pore size usually means a higher surface area/volume ratio, thus favoring cell adhesion to the scaffold and promoting bone tissue regeneration [46], [47], [48]. Previous studies have shown that the quality of bone ingrowth into porous systems is determined by their pore sizes [49], [50], [51]. The optimal pore size for mineralized bone ingrowth still seems to be a controversial topic. Previous studies concluded that the pore size should be larger than 100 µm for regenerating mineralized bone [52], while another group showed that there is no threshold value for new bone ingrowth with pore sizes ranging from 50 to 125 mm under nonload-bearing conditions [49]. The porous structure of our scaffolds was achieved by a freeze-drying method. SEM results showed that the average diameter of the micro-pores generated in the CAH scaffold was 100–110 µm. The porosity of various scaffolds has been evaluated using a liquid displacement method. The porosity of the CAH scaffold was determined to about 74.54%. Our in vivo studies further showed that CAH scaffold could lead to effective cell proliferation and bone ingrowth, suggesting that a CAH scaffold with a 100–110 µm pore size is suitable for bone ingrowth.

XRD and EDX analysis of the synthesized CAH specimens showed the crystalline peaks typical for each component. But the peaks became broader and weaker compared to the standard spectrum, due to molecular interactions between each component that influenced the diffraction peaks [29], [53]. The Ca/P ratio in the CAH scaffold is 1.60, which is slightly lower than pure HA. This might be because of the presence of other ions, such as Na+, and K+. Small amounts of these ions can substitute for calcium ions in the crystal lattice, resulting in a lower Ca/P ratio [54].XRD spectra also indicated that the peaks associated with HA are broad, which is indicative of poor crystallinity. Previous studies that generated HA with Ca/P ratios less than 1.67 [54], [55] showed that HA with lower crystallinity have greater potential for resorption in vivo compared to highly crystalline HA, which resorbs very slowly [56]. Zhang et al prepared a calcium-deficient HA scaffold (ca/p ratio = 1.5), with 40wt% of hydroxyapatite, and implanted it in the defects of rabbits for 3 months. This might explain why there was little material left 12 weeks after the implantation.

In bone tissue engineering, BMPs are known to play an important role [57]. They are essential in various stage of bone healing, from the initial phases of fracture repair to later stages of osteogenesis. But, since it has a short half-life, delivery of BMP-2 alone to a defect results in its rapid clearance [58]. Previous studies have demonstrated that adenovirus-mediated BMP gene therapy can lead to harmful side effects such as tumorigenesis and immunogenicity [18], [59]. By using biomaterials as carriers, they may provide controlled and sustained delivery of a growth factor, retaining BMP-2 at the defect site and mimicking its temporal profile during bone healing in vivo [60]. Hence strategies such as implantation of BMP-2 together with alginate, which is considered a superior carrier for BMPs, as a delivery system [61], [62]. Type I Col-gel, which is widely used as a drug delivery system, was employed as a control in these studies. ELISA results showed that BMP-2 exhibited a burst release in the Col-B2 group, its level dropping rapidly in 7 days, while the CAH/B2 group maintained the concentration in a higher level and the prolonged release of BMP-2 continued for 21 days, suggesting that the release velocity from the CAH/B2 scaffold manifested a sustained release of BMP-2 and led to osteogenic differentiation of the MSCs. The in vitro release data in our experiment are probably not indicative of in vivo release rates, which could be faster due to higher enzyme activity activities. Given the observation that in vitro and in vivo release often show different profiles [63], we can nevertheless estimate the total dose that was available in the implanted constructs. Many different biomaterials are suitable for controlled release. Alginate is a well-known agent for growth factor delivery, characterized by a small initial burst and leaving no tissue damaging residual material. As such, alginate is used in many FDA-approved devices. Our histology analysis showed there was very little residual material left, indicating that BMP-2 might be released from CAH/B2 scaffolds as the result of a combination of diffusion and scaffold degradation.

The ALP activity and the level of calcium deposition are important considerations for evaluating osteoblast differentiation. Our results showed ALP activity increased after 1, 2 and 3 weeks and the level of calcium mineral deposition in the CAH/B2 group was also increased 2.8 fold after 3 weeks compared with that in the CAH group. Furthermore, qRT-PCR results showed that OCN, OPN and Col1α1 mRNA expression increased in the CAH/B2 group. These data suggest that BMP-2, when delivered in a CAH scaffold, still retains its biological ability. CAH/B2 scaffolds provide an effective approach for BMP-2 delivery as well as enhanced ALP activity, conferring a stimulatory effect on the differentiation of osteoblastic cells and matrix mineralization.

In our in vivo study, MSCs were seeded on a CAH/B2 scaffold and set into a well-established critical sized rat calvarial bone defect. The results from densitometric scans showed that BMD in the CAH/B2+M group was much higher than in the other groups. The CAH/B2+M group exhibited robust osteogenic activity, with complete closure of the bony defects. Histological analysis complemented the BMD result. These results demonstrate that CAH/B2 scaffolds are biodegradable and biocompatible. CAH scaffolds enhanced defect closure and mineralization compared to untreated control defects, indicating CAH scaffolds are osteoconductive, while CAH/B2 scaffolds can provide an even more effective approach to the repair of a critical sized bone defect. Notably, the critical sized defects were repaired much better in the MSC containing groups compared with the group without MSCs, and exhibited no sign of rejection in any group, indicating that at least most of the implanted allogeneic MSCs maintained their viability and apparently suffered no immunorejection by the host. This conclusion is supported by some previous findings [64], [65]. In addition, the results of this study support the concept that BMP2 can enhance bone regeneration. Localized and sustained BMP2 delivery from CAH/B2 significantly increased the expression of osteoblast marker genes and promoted bone formation in the bone defect area. Hence, the effects of BMP2 delivery in CAH/B2 observed in this study corroborate previous work, highlighting the importance of the CAH/B2 scaffold and MSCs in bone healing.

In conclusion, this study provides the first evidence that this CAH/B2 scaffold is a good carrier for BMP-2 and an efficient vehicle for stem cells to promote new bone formation. The combination of the CAH/B2 scaffold with MSCs can dramatically enhance new bone formation and lead to a nearly complete repair of critical sized calvarial bone defects. On the basis of the data presented here, it appears that CAH scaffolds could be used for the repair of bone defects and functional bone tissue engineering applications. The use of osteogenically differentiated MSCs and a combination of MSCs and BMP-2 may further enhance osteogenesis.
